# Prognostic Value of ctDNA Mutation in Melanoma: A Meta-Analysis

**DOI:** 10.1155/2021/6660571

**Published:** 2021-05-04

**Authors:** Yang Zheng, Hongyan Sun, Lele Cong, Chenlu Liu, Qian Sun, Nan Wu, Xianling Cong

**Affiliations:** ^1^Department of Dermatology, China-Japan Union Hospital of Jilin University, Changchun, China; ^2^Biobank, China-Japan Union Hospital of Jilin University, Changchun, China

## Abstract

**Purpose:**

Melanoma is the most aggressive form of skin cancer. Circulating tumor DNA (ctDNA) is a diagnostic and prognostic marker of melanoma. However, whether ctDNA mutations can independently predict survival remains controversial. This meta-analysis assessed the prognostic value of the presence or change in ctDNA mutations in melanoma patients.

**Methods:**

We identified studies from the PubMed, EMBASE, Web of Science, and Cochrane databases. We estimated the combined hazard ratios (HRs) for overall survival (OS) and progression-free survival (PFS) using either fixed-effect or random-effect models based on heterogeneity.

**Results:**

Sixteen studies including 1,781 patients were included. Both baseline and posttreatment detectable ctDNA were associated with poor OS (baseline detectable vs. undetectable, pooled HR = 1.97, 95% CI = 1.64–2.36, *P* < 0.00001; baseline undetectable vs. detectable, pooled HR = 0.19, 95% CI = 0.11–0.36, *P* < 0.00001; posttreatment detectable vs. undetectable, pooled HR = 2.36, 95% CI = 1.30–4.28, *P*=0.005). For PFS, baseline detectable ctDNA may be associated with adverse PFS (baseline detectable vs. undetectable, pooled HR = 1.41, 95% CI = 0.84–2.37, *P*=0.19; baseline undetectable vs. detectable, pooled HR = 0.43, 95% CI = 0.19–0.95, *P*=0.04) and baseline high ctDNA and increased ctDNA were significantly associated with adverse PFS (baseline high vs. low/undetectable, pooled HR = 3.29, 95% CI = 1.73–6.25, *P*=0.0003; increase vs. decrease, pooled HR = 4.48, 95% CI = 2.45–8.17, *P* < 0.00001). The baseline BRAF^V600^ ctDNA mutation-positive group was significantly associated with adverse OS compared with the baseline ctDNA-negative group (pooled HR = 1.90, 95% CI = 1.58–2.29, *P* < 0.00001). There were no significant differences in PFS between the baseline BRAF^V600^ ctDNA mutation-detectable group and the undetectable group (pooled HR = 1.02, 95% CI = 0.72–1.44, *P*=0.92).

**Conclusion:**

The presence or elevation of ctDNA mutation or BRAF^V600^ ctDNA mutation was significantly associated with worse prognosis in melanoma patients.

## 1. Introduction

Melanoma is the most aggressive form of skin cancer, originating from pigment-producing melanocytes. Though it accounts for only 10% of all skin cancers, it is responsible for more than 80% of skin cancer-related deaths. The development of targeted therapy (MAPK pathway inhibitors) [1, 2] and immunotherapy (checkpoint inhibitors) [3–5] has led to substantial improvements in overall survival (OS). However, a significant number of patients develop acquired resistance or do not benefit from therapy and, in some cases, therapy can be toxic [6, 7]. Therefore, the monitoring of disease progression and prognosis of patients are of vital importance and contribute to improving the quality of life of melanoma patients.

In recent years, many studies have focused on the value of circulating tumor DNA (ctDNA) in monitoring disease status and prognosis [8–12]. Some studies have focused on the prognostic value of ctDNA in melanoma patients, although the results are controversial. A study [13] conducted by Tan et al. confirmed that ctDNA detection before surgery and postoperatively can identify melanoma patients at highest risk of relapse, although there was no significant association between detectable ctDNA (at baseline or postoperatively) and OS. Lee et al. [14] proposed that OS was significantly worse for patients with detectable ctDNA postsurgery and high-risk stage II/III melanoma. In the study [15] by Seremet et al., undetectable ctDNA at baseline remained significantly correlated with progression-free survival (PFS) and OS in metastatic melanoma patients treated with anti-PD1 therapy. Forthun et al. conducted a study [16] of bevacizumab in the treatment of metastatic malignant melanoma and showed that  ≤1% BRAF/NRAS-positive ctDNA before and during treatment reflected a positive response to therapy, through an increase in the durations of PFS and OS. A study by Board et al. [17] indicated that cfDNA BRAF detection is not associated with poorer PFS in stage III/IV advanced melanoma. Besides, there is still a lack of systematic evidence to prove the prognostic value of ctDNA in melanoma patients. Sequencing data identified melanoma as the most frequently mutated tumor type analyzed by The Cancer Genome Atlas (TCGA) [18]. Therefore, the aim of this meta-analysis was to evaluate the prognostic significance of ctDNA mutations in melanoma patients, in terms of OS and PFS.

## 2. Methods

### 2.1. Data Sources and Searches

We conducted systematic searches of the PubMed, EMBASE, Web of Science, and Cochrane databases for entries up until March, 2020 without language or study period restrictions. The search terms included (“ctDNA” or “circulating tumor DNA” or “circulating DNA” or “free DNA” or “cell free DNA” or “plasma DNA” or “serum DNA”) and (“Melanoma” or “Skin cancer” or “Skin neoplasia” or “skin neoplasm”) and (“prediction” or “outcome” or “predictive” or “prognosis” or “prognostic”). A manual search of related articles and references cited in these articles was performed to identify all available studies.

### 2.2. Inclusion and Exclusion Criteria

Studies were included in the analysis if they met the following criteria: (1) all patients enrolled in the study were diagnosed with melanoma, (2) ctDNA mutation was assessed using plasma or serum, (3) endpoints included PFS or OS, and sufficient data were presented for determining or calculating the hazard ratio (HR) and 95% confidence interval (95% CI).

Studies were excluded if (1) fewer than 15 patients were included in the statistical analysis of prognosis value of ctDNA, (2) samples were not drawn from peripheral blood (e.g., they came from cerebrospinal fluid or lymphatic fluid), and (3) nonresearch publications such as editorials, reviews, and letters, or the publications were duplicated articles.

### 2.3. Data Extraction and Quality Assessment

Two investigators independently reviewed all eligible studies and extracted the following information: first author name, publication year, country, number of patients analyzed, tumor stage, sample origin, time of sample collection, clinical therapy, method/platform of ctDNA detection, target genes/variants, cutoff value, number of experimental/control samples, positive ratio, and endpoint, and follow-up duration and confounding factors, if provided. When the article had multiple queues or groups of patients, and an HR for survival curve was provided for each queues/groups, results of all these queues/groups were recorded as independent data. The quality of all included studies was evaluated using the Newcastle–Ottawa Quality Assessment Scale (NOS) [19]. An NOS score of 5–9 stars was considered indicative of a high quality meta-analysis.

### 2.4. Statistical Analysis

Hazard ratios (HRs) and their 95% CIs for PFS and OS were recorded to clarify the prognostic value of ctDNA. For studies in which HRs and 95% CIs were not available, we extracted survival rates from Kaplan–Meier curves by using Engauge Digitizer version 4.1 [20]. To assess the heterogeneity among studies, pooled HRs were initially calculated using a fixed effects model. If there was significant heterogeneity among studies (*I*^2^ > 50%), the random effects model was adopted [21]. *I*^2^ > 50% and *P* < 0.05 were considered significant for heterogeneity.

For forest plots with more than 10 included studies or results, we evaluated publication bias using funnel plots for visual inspection and conducted quantitative estimations using Egger's test. Sensitivity analysis was performed by excluding each study in turn to assess the stability of the results. All analyses were carried out using Review Manager version 5.3 (The Cochrane Collaboration, Copenhagen: The Nordic Cochrane Center, 2012) and STATA version 12.0 (STATA Corporation, College Station, TX, USA).

## 3. Results

### 3.1. Search Results

**Figure 1:**
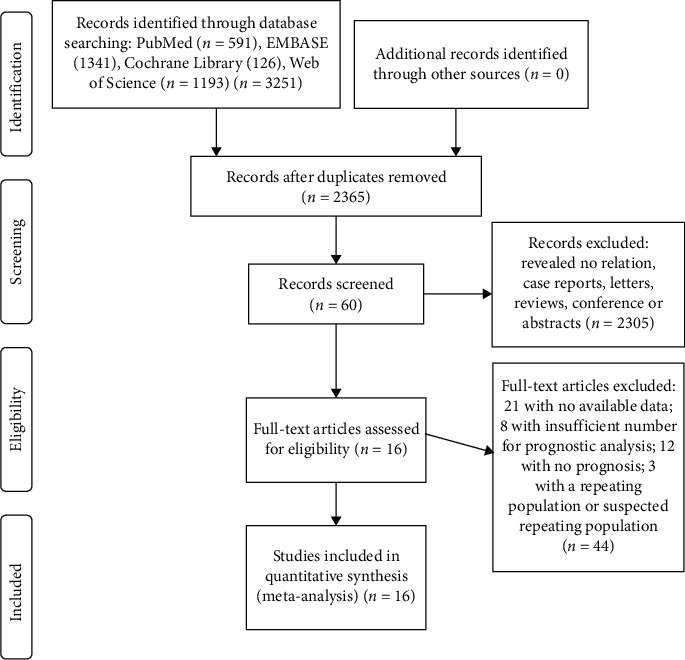
Study selection strategy and flow diagram.

A total of 2,365 articles were identified after removing duplicates. By reviewing titles and abstracts, 2,305 articles were excluded, of which 2,114 were not related to the disease or subject of our meta-analysis, 86 were reviews or systematic evaluations, and 105 were abstracts, conference papers, or case reports. After detailed reading and evaluation of 60 articles, we excluded eight articles because the number of patients analyzed for prognosis was less than 15. Twelve articles were excluded for lack of prognostic information. Twenty-one articles were excluded because they did not provide sufficient data to extract HRs for PFS or OS. Further three articles with or suspected to have overlapping study populations were excluded. Finally, 16 articles [12–17, 22–31] proved eligible for inclusion and were analyzed (Figure 1).

### 3.2. Literature Characteristics and Quality

The characteristics and quality of studies included in the meta-analysis are described in Table 1. The 16 studies [12–17, 22–31] were published between 2007 and 2020, and the sample size of ctDNA prognosis analyses ranged from 20 to 551, with an overall total of 1,781. Among the studies, four [12, 13, 25, 29] were from Australia, and one study each was from the UK [14], Poland [22], Spain [28], Norway [16], Italy [24], Belgium [15], France [27], and USA [31]. In addition, one study [23] was from Belgium and Germany. The samples from one study [17] were obtained from the participants of a phase 2 clinical trial in which the patients were from 10 countries. The samples from one study [26] were obtained from the participants of a phase 3 clinical trial in which the patients were from 12 countries. One study [30] included samples from the participants of four clinical trials, each involving patients from more than one country. Since two of the clinical trials enrolled the same patient populations, we chose the one with larger sample size for meta-analysis. In addition to the study [30] from three clinical trials, another three studies [12, 23, 26] were grouped by cohort or drug therapy. Therefore, we considered them as independent studies. All the patients in 15 studies had advanced melanoma, while the patients in one study [14] were at stage II-III. Plasma ctDNA levels were assessed in 12 studies [12–16, 22, 23, 25–27, 29, 30], serum ctDNA levels were assessed in three studies [17, 24, 31], and ctDNA was extracted from serum and plasma samples in one study [28]. Twelve studies [12, 13, 15, 16, 22–24, 26–29, 31] analyzed ctDNA from blood samples before and after treatment, and four studies [14, 17, 25, 30] analyzed ctDNA before treatment. However, among these 12 studies [12, 13, 15, 16, 22–24, 26–29, 31], PFS or OS analysis was not performed in eight studies [12, 15, 22, 24, 26–29] using posttreatment data, and pretreatment data was not performed in one study [31]. Droplet digital PCR (ddPCR) was used to detect ctDNA in blood samples in 11 studies [12–16, 22–26, 29]. Only the BRAF^V600^ ctDNA mutation was detected in seven studies [17, 22, 24, 26, 28, 30, 31], and multiple genes were detected in another study [23], although PFS or OS analysis of the BRAF^V600^ mutation was available. The other studies [12–16, 25, 27, 29] all detected the mutations in multiple genes. The quality of studies included was evaluated by NOS, and all the studies received at least six stars, all of which were regarded as high quality.

### 3.3. Prognostic Value of ctDNA in Melanoma

#### 3.3.1. Meta-Analysis of ctDNA Predicting OS

**Figure 2:**
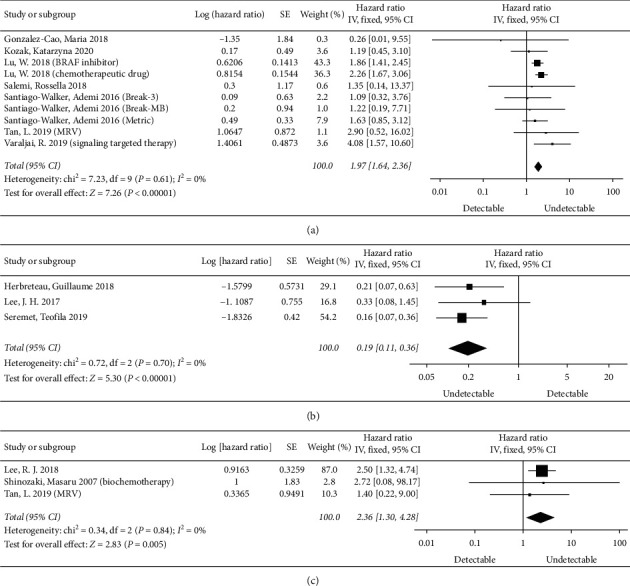
Meta-analysis of ctDNA predicting OS. (a) Effect of baseline ctDNA on OS in melanoma (detectable vs. undetectable). (b) Effect of baseline ctDNA on OS in melanoma (undetectable vs. detectable). (c) Effect of posttreatment ctDNA on OS in melanoma (detectable vs. undetectable).

A total of 11 studies [13, 15, 16, 22–24, 26–30] assessed the relationship between baseline ctDNA and OS. Seven studies (10 results) [13, 22–24, 26, 28, 30] analyzed the effect of baseline detectable ctDNA on OS compared with baseline undetectable ctDNA. The multivariate results of one study [22] analyzing the effect of the log-transformed concentration of baseline ctDNA on prognosis were not included in the analysis. As shown in Figure 2(a), the risk was significantly higher in the baseline ctDNA-positive group than in the baseline ctDNA-negative group in terms of mortality (pooled HR = 1.97, 95% CI = 1.64–2.36, *P* < 0.00001). The *I*^2^ statistical heterogeneity was not significant (*I*^2^ = 0%). This suggests that baseline detectable ctDNA is associated with adverse OS.

Three studies [15, 27, 29] indicated the association of baseline undetectable ctDNA with OS compared with baseline detectable ctDNA. Our results showed that, in melanoma, the risk of mortality in the baseline undetectable ctDNA group was significantly lower than that in the baseline detectable ctDNA group (pooled HR = 0.19, 95% CI = 0.11–0.36, *P* < 0.00001), with no heterogeneity (*I*^2^ = 0%) (Figure 2(b)). This suggests that baseline undetectable ctDNA is associated with better OS.

In addition, one study [16] analyzed the relationship between baseline high ctDNA and OS compared with baseline undetectable or low ctDNA, suggesting that baseline high ctDNA was associated with adverse OS. One study [28] suggested that baseline undetectable or low ctDNA was significantly associated with better OS compared with baseline high ctDNA. One study [26] also showed a relationship between high versus low, high versus undetectable, and low versus undetectable and OS, with statistically significant differences. Another study [29] analyzed patients with posttreatment negative-ctDNA and baseline undetectable ctDNA was significantly associated with better OS compared with baseline detectable ctDNA. The above results were not included in the analysis due to different classification methods. These results suggest that baseline detectable ctDNA is associated with adverse OS.

Four studies [13, 14, 16, 31] evaluated the relationship between posttreatment ctDNA and OS, although one [16] was not included in the analysis because of the different grouping method. Our results showed that in melanoma, compared with the posttreatment undetectable ctDNA group, the posttreatment detectable ctDNA group was associated with adverse OS (pooled HR = 2.36, 95% CI = 1.30–4.28, *P*=0.005), with no heterogeneity (*I*^2^ = 0%) (Figure 2(c)). One study [16] that was not included in the analysis examined the relationship between posttreatment high ctDNA and OS compared with posttreatment undetectable and low ctDNA, suggesting that posttreatment high ctDNA was significantly associated with adverse OS. These results suggest that posttreatment detectable ctDNA is associated with adverse OS.

#### 3.3.2. Meta-Analysis of ctDNA Predicting PFS

**Figure 3:**
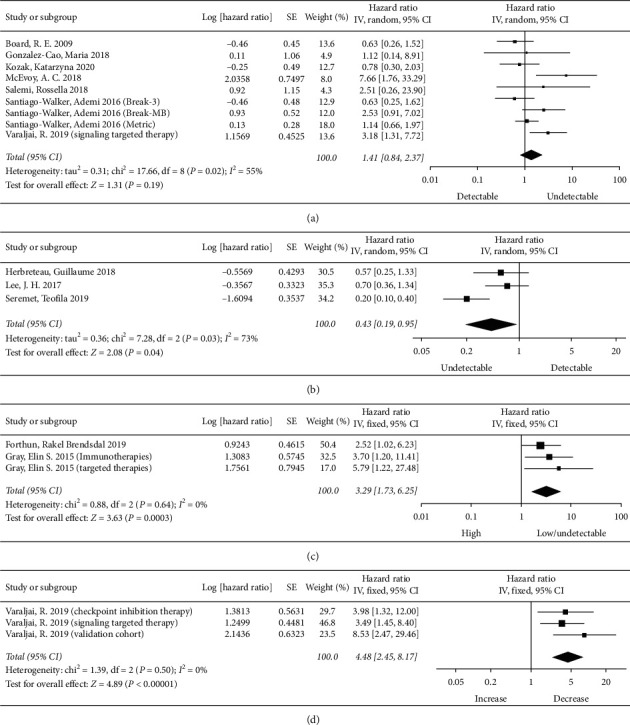
Meta-analysis of ctDNA predicting PFS. (a) Effect of baseline ctDNA on PFS in melanoma (detectable vs. undetectable). (b) Effect of baseline ctDNA on PFS in melanoma (undetectable vs. detectable). (c) Effect of baseline ctDNA on PFS in melanoma (high vs. low/undetectable). (d) Effect of ctDNA change on PFS in melanoma (increase vs. decrease).

A total of 12 studies [12, 15–17, 22–25, 27–30] assessed the relationship between baseline ctDNA and PFS. Seven studies (nine results) [17, 22–25, 28, 30] analyzed the effect of baseline detectable ctDNA on PFS compared with baseline undetectable ctDNA. The multivariate results of one study [22] analyzing the effect of the log-transformed concentration of baseline ctDNA on prognosis were not included in the analysis. As shown in Figure 3(a), the baseline detectable ctDNA group benefited less than the baseline undetectable ctDNA group in terms of treatment response, although the differences were not statistically significant (pooled HR = 1.41, 95% CI = 0.84–2.37, *P*=0.19). The *I*^2^ statistical heterogeneity was high (*I*^2^ = 55%); thus, a random-effects model was used.

Three studies [15, 27, 29] indicated the association of baseline undetectable ctDNA with PFS compared with baseline detectable ctDNA. The results showed that baseline undetectable ctDNA was significantly associated with better PFS (pooled HR = 0.43, 95% CI = 0.19–0.95, *P*=0.04, Figure 3(b)). The *I*^2^ statistical heterogeneity was high (*I*^2^ = 73%); thus, a random-effects model was used.

Two studies (three results) [12, 16] analyzed the differences between the baseline high ctDNA group and the baseline undetectable or low ctDNA group and evaluated PFS. The baseline high ctDNA group benefited less than the baseline undetectable or low ctDNA group in terms of treatment response (pooled HR = 3.29, 95% CI = 1.73–6.25, *P*=0.0003), with no heterogeneity (*I*^2^ = 0%) (Figure 3(c)).

In addition, data provided in two studies [28, 29] not included in the above analysis due to different grouping methods showed that baseline undetectable or low ctDNA was significantly associated with better PFS compared with baseline high ctDNA [28] and that in patients with posttreatment negative-ctDNA, baseline undetectable ctDNA was significantly associated with better PFS compared with baseline detectable ctDNA [29]. These results suggest that baseline detectable ctDNA may be associated with adverse PFS.

The relationship between posttreatment ctDNA detection and PFS was analyzed in one study [16], which suggested that posttreatment high ctDNA was significantly associated with adverse PFS compared with posttreatment undetectable or low ctDNA. Due to the insufficient number of studies, no analysis was carried out.

One study (three results) [23] indicated that the association of ctDNA changed with PFS. The increased ctDNA group had less therapeutic benefit than the decreased ctDNA group (pooled HR = 4.48, 95% CI = 2.45–8.17, *P* < 0.00001), with no heterogeneity (*I*^2^ = 0%) (Figure 3(d)).

### 3.4. Prognostic Value of the ctDNA BRAF^V600^ Mutation in Melanoma

**Figure 4:**
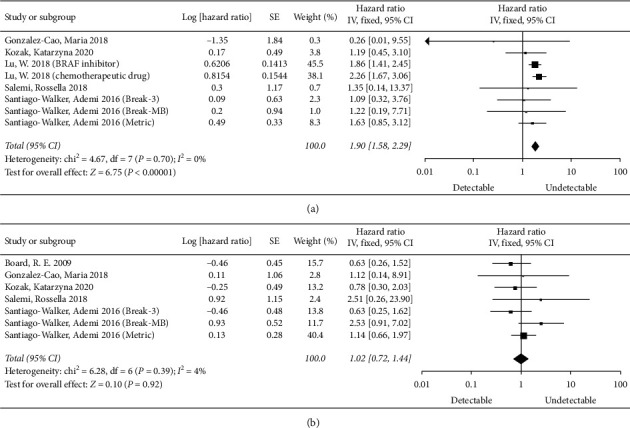
Meta-analysis of the BRAF^V600^ ctDNA mutation predicting OS or PFS. (a) Effect of baseline BRAF^V600^ ctDNA mutation on OS in melanoma (detectable vs. undetectable). (b) Effect of baseline BRAF^V600^ ctDNA mutation on PFS in melanoma (detectable vs. undetectable).

We only further analyzed the effects of baseline BRAF^V600^ mutation detectable ctDNA on PFS and OS, compared with baseline BRAF^V600^ mutation undetectable ctDNA because of different grouping methods and the limited number of studies. Our results showed that the risk was significantly higher in the baseline BRAF^V600^ ctDNA mutation-positive group than in the baseline ctDNA-negative group in terms of mortality (pooled HR = 1.90, 95% CI = 1.58–2.29, *P* < 0.00001), with no heterogeneity (*I*^2^ = 0%) (Figure 4(a)). The differences between the baseline BRAF^V600^ ctDNA mutation detectable group and undetectable group were not statistically significant in terms of treatment response (pooled HR = 1.02, 95% CI = 0.72–1.44, *P*=0.92), with low heterogeneity (*I*^2^ = 4%) (Figure 4(b)).

### 3.5. Heterogeneity Analysis

A subgroup analysis was performed on the forest plot with the largest number of studies to identify the factors that may influence interstudy heterogeneity, including the effects of baseline detectable ctDNA on PFS and OS, compared with baseline undetectable ctDNA.

Table S1 shows the results of subgroup analyses, stratified by target gene, sample origin, method, and data source. There was no heterogeneity among the subgroups that assessed OS (*I*^2^ = 0%). In most subgroups, baseline detectable ctDNA was significantly associated with adverse OS, although there was no significant correlation between baseline detectable ctDNA and OS in the non-ddPCR ctDNA evaluation subgroup and in the subgroup whose data were extracted from the survival curve (pooled HR = 1.41, 95% CI = 0.82–2.43, *P*=0.21; pooled HR = 1.36, 95% CI = 0.85–2.15, *P*=0.20, respectively). For the subgroups that evaluated PFS, heterogeneity was significantly reduced according to whether the target gene included only BRAF^V600^ or not, and according to whether the data was directly provided by the studies or extracted from the survival curve. As it happened, the two subgroups included the same studies, whose target genes were BRAF^V600^, and data were extracted from the survival curve, which showed no significant correlation between baseline detectable ctDNA and PFS (pooled HR = 1.02, 95% CI = 0.72–1.44, *P*=0.92, *I*^2^ = 4%). Meanwhile, the subgroup whose data was provided directly by the studies suggested that baseline detectable ctDNA was associated with adverse PFS (pooled HR = 4.02, 95% CI = 1.88–8.59, *P*=0.0003, *I*^2^ = 1%). These results suggest that the sources of heterogeneity may be the method of ctDNA assessment, target genes, and data sources.

### 3.6. Sensitivity Analysis

Sensitivity analysis based on the change in the combined effect size by excluding the included studies one by one is an important method mainly used to evaluate the robustness and reliability of the combined results of meta-analysis. After sensitivity analysis, the overall effect sizes of the two meta-analyses were significantly modified. Among them, one study [14] had a significant impact on the effect of posttreatment detectable ctDNA on OS. After excluding this study, the overall HR changed from 2.36 (95% CI = 1.30–4.28, *P*=0.005) to 1.61 (95% CI = 0.31–8.40, *P*=0.57), and the heterogeneity remained at 0. Two studies [15, 27] had a significant impact on the effect of baseline undetectable ctDNA on PFS. After excluding one study [27], the overall HR changed from 0.43 (95% CI = 0.19–0.95, *P*=0.04) to 0.38 (95% CI = 0.11–1.28, *P*=0.12) and the heterogeneity was almost unchanged (from *I*^2^ = 73% to *I*^2^ = 85%). After excluding the other study [15], the overall HR changed to 0.65 (95% CI = 0.39–1.09, *P*=0.10) and the heterogeneity decreased to 0.

### 3.7. Publication Bias

**Figure 5:**
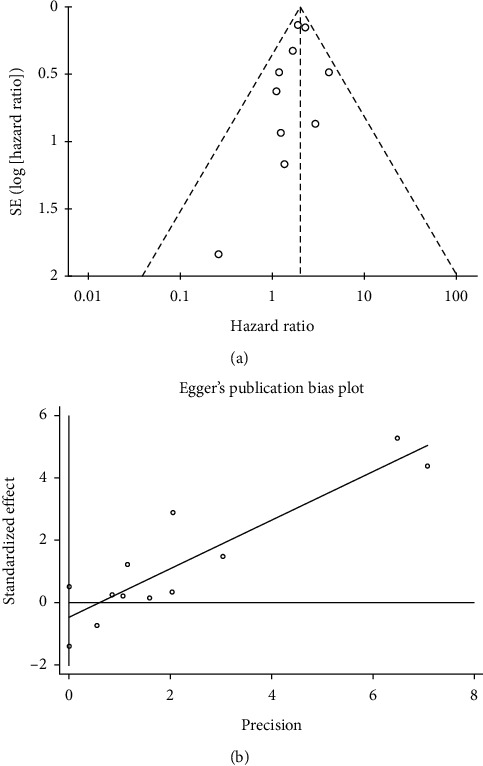
Funnel plot and Egger's test of meta-analysis for the association between baseline ctDNA and OS.

Funnel plots and Egger's test were used to assess publication bias for forest plots containing more than 10 studies or results, thus assessing the impact of baseline detectable ctDNA on OS. The results revealed no evidence of significant publication bias (Egger's test, *P*=0.306) (Figure 5).

## 4. Discussion

In 2012, melanoma was the 15th most common cancers worldwide [32]. Unfortunately, the worldwide incidence of cutaneous melanoma has been increasing at a faster rate each year than that of any other type of cancer [33]. The treatment of melanoma has been revolutionized in the past decade. In particular, the PFS and OS in melanoma patients have improved significantly with the introduction of immune checkpoint inhibitors and the new selective tyrosine kinase inhibitors, including the BRAF and MEK inhibitors [34], although there are still a certain proportion of patients experiencing early tumor recurrence or progression. As a result, major efforts have been made to better monitor the evolution of the disease and the prognosis of patients, such as considering histopathological features, patient characteristics, biochemical indicators, and genetic mutations [33]. Sequencing data has shown that melanoma has a median mutation rate of 10 mutations/Mb, the highest of all cancers so far analyzed by the TCGA network [18]. Meta-analysis has demonstrated that BRAF mutation is an absolute risk factor for survival in melanoma patients [35]. However, it is difficult to study genomic changes in repeated tumor biopsies during treatment. Since Mandel and Metais [36] first discovered circulating nucleic acids in the blood of healthy humans in 1948, ctDNA has been widely used in many disciplines. Analysis of ctDNA can provide a noninvasive method to assess prognosis and response to treatment [12]. Our meta-analysis assessed the prognostic significance of ctDNA mutation in baseline or treatment samples from melanoma patients and played a guiding role in the clinical treatment of melanoma.

This meta-analysis included 16 studies including 1,781 melanoma patients for a prognostic analysis. We analyzed differences in OS and PFS at the endpoint of observation for mutations in ctDNA at baseline and after treatment. We also calculated the relevant HRs and 95% CIs. The results revealed that ctDNA mutation was significantly associated with the prognosis of melanoma patients. Specifically, patients with detectable ctDNA mutation tended to have adverse OS compared to patients where ctDNA mutation was not detected, either at baseline or after treatment. Patients with low or undetectable ctDNA mutation at baseline tend to have better PFS compared with patients with high ctDNA mutation, and baseline detectable ctDNA may be associated with adverse PFS. Patients with decreased ctDNA levels tend to have favorable PFS compared with patients with increased ctDNA levels. CtDNA BRAF^V600^ mutation is also a prognostic biomarker with similar prognostic value. Patients with detectable BRAF^V600^ ctDNA at baseline tend to have worse OS compared to patients with undetectable BRAF^V600^ ctDNA, while baseline detectable BRAF^V600^ ctDNA may be associated with worse PFS.

The result of our subgroup analysis stratified by target gene showed that BRAF^V600^ mutation was significantly associated with worse OS, while patients with BRAF^V600^ ctDNA tended to have poor PFS, although without statistical significance. However, the subgroups in which the association between multiple gene ctDNA mutations and PFS or OS was analyzed included one study each; therefore, the results of the subgroup analysis were not representative or comparable due to the differences in tumor stage, treatment, and method of ctDNA assessment in the two studies. Melanoma has the highest somatic cell mutation rate [37] and a wide range of genomic alterations. BRAF mutations occur frequently in up to 66% of patients with melanoma [38], while these activation mutations in BRAF lead to constitutive activation of kinase function independent of upstream signaling from RAS, ultimately promoting cell growth and inhibiting cell apoptosis [39]. NRAS mutations occur in more than 20% of patients with cutaneous melanoma [40], leading to the activation of MAPK, PI3K, and other cellular signaling pathways, and resulting in cell growth, proliferation, and cell cycle dysfunction. According to the data published by the TCGA network based on whole exome sequence analysis of patients with primary and/or metastatic melanoma, melanoma could be classified into four genomic subtypes: mutant BRAF, mutant RAS, mutant NF1, and Triple-WT (wild-type) [40]. The different genomic subtypes may be of predictive value given the therapeutic targets currently available [41]. Therefore, multigene ctDNA mutation detection can better predict the prognosis of patients with melanoma.

The results of the subgroup analysis classified by method of ctDNA assessment showed that ddPCR was more effective than other methods. The content of ctDNA in peripheral blood is very small, and the content and nucleic acid polymorphisms in different patients vary greatly [42]. Low-sensitivity methods will inevitably miss some of the mutated ctDNA in patients' peripheral blood and produce false negative results. Therefore, the detection of ctDNA requires highly specific and sensitive techniques. DdPCR improves on the advantages of standard PCR, improves accuracy, and reduces error rates [43]. Furthermore, it is cost effective and rapid, allowing highly sensitive detection of allelic variants with a resolution of 0.005% and simultaneously detecting four different mutations [44]. Although this technique allows the detection of low-frequency mutations, preidentification of target genes is required. The high frequency of BRAF^V600^ or NRAS^Q61^ mutations in patients with melanoma enhances the effectiveness of ddPCR in a majority of the cases [45].

The results of subgroup analysis stratified by sample sources were consistent with the conclusions of Meddeb et al. [46] in that plasma was a better source of ctDNA than serum, this being because, during serum isolation, normal DNA derived from leukocyte lysis during coagulation is much lower than from plasma [47].

There are many biomarkers for the prognosis and prediction of melanoma used clinically. The 8th edition American Joint Committee on Cancer (AJCC) melanoma staging system [48] determined that tumor thickness, mitotic rate, and ulceration were the most dominant prognostic factors. In addition, the only circulating biomarker with significant prognostic value is lactate dehydrogenase (LDH). There are several other circulating biomarkers with diagnostic and prognostic value, such as C-reactive protein (CRP), S100 protein (S100B), melanoma inhibitory activity (MIA), etc. However, the poor sensitivity or specificity of these markers seriously limits their routine use in melanoma [49]. CtDNA is highly fragmented DNA that is distributed by tumor cells into the circulation [50]. A study [51] has shown that ctDNA carries genetic information from the entire tumor genome and is a widely applicable, sensitive, and specific biomarker, thus providing insight into the clonal heterogeneity and evolution of all solid cancers at any time. Our meta-analysis clearly indicated the detection of ctDNA BRAF^V600^ and other gene mutations has shown significant value in predicting treatment response and outcome in melanoma. In addition to detecting mutated ctDNA, epigenetic changes and loss of heterozygosity (LOH) of ctDNA can also be detected and analyzed. Two studies by Mori et al. [52, 53] demonstrate the utility of detecting circulating methylated tumor-related genes as a potentially predictive marker of overall survival, and hypermethylation of estrogen receptor *α* predicting progression-free and overall survival. Due to the small number of relevant studies, we did not conduct a meta-analysis.

In the present meta-analysis, all patients in most of the studies had advanced melanoma, while the patients in one study were at stage II-III. Therefore, our conclusions are applicable to patients with advanced melanoma. Due to the limited number of studies, no subgroup analysis of tumor-node-metastasis (TNM) staging was performed. In addition, several limitations in this study should be addressed. We may not have identified published or unpublished studies with negative results, and some studies had a short follow-up time or small sample sizes; therefore, the results should be interpreted with caution. In addition, we hypothesized that possible sources of bias are as follows, and these factors may also lead to heterogeneity: (1) most studies only used univariate analysis, and the size of effect values may be overestimated compared with multivariate analysis; (2) multivariate analysis was used in some studies to obtain accurate estimates, although the confounders used in multivariate analysis were different, which may affect HR values; (3) in most studies, multiple gene ctDNA mutations were detected, which were not identical and may have introduced bias; (4) not all HRs and 95% CIs included in the meta-analysis were directly collected from the original articles; thus, we extracted survival rates from Kaplan–Meier curves by using Engauge Digitizer version 4.1 [20] to reduce deviation; and (5) follow-up time and cutoff values were not consistent, which may have also caused deviations in the results.

## 5. Conclusion

Despite these limitations, our meta-analysis clearly indicates the prognostic value of ctDNA mutations in melanoma; that is, the presence or elevation of ctDNA mutation or BRAF^V600^ ctDNA mutation was significantly associated with worse prognosis in melanoma patients. However, in view of some of the limitations of this study, the results of our meta-analysis need to be verified by further research. With further exploration and understanding of ctDNA biological function and clinical value, as well as the validation of more large-scale, high-quality prospective studies, ctDNA could eventually be applied in clinical practice and may eventually become part of routine melanoma staging.

## Figures and Tables

**Table 1:** The detailed characteristics of the included studies.

First author, publication year	Country	Patient no. or prognostic analysis no./total no.	TNM stage	Sample origin	Time of sample collection	Clinical therapy	Detection methods	Target genes/variants	Cutoff value	Experimental no./control no.	Positive ratio	Endpoint	Follow-up duration	Confounding factors	NOS score
Kozak et al., [22]	Poland	62	IIIC-IV	Plasma	Pretreatment, posttreatment^*∗*^	BRAF inhibitor vemurafenib	ddPCR (bio-rad)	BRAF^V600E^	NA	48/14 (pretreatment), 18/44 (posttreatment)^*∗*^	48/62 (pretreatment), 18/62 (posttreatment)^*∗*^	PFS, OS	55.4 months (median)	—	7
Váraljai et al., [23] (immune checkpoint inhibition therapy)	German	18/33	III-IV	Plasma	Pretreatment, posttreatment	Immune checkpoint inhibitors	ddPCR	BRAF^V600E^, NRAS^Q61^, TERT^PROM^	NA	12/6^m^ (BRAF^V600E^)	NA	PFS	NA	—	6
Váraljai et al., [23] (signaling-targeted therapy)	German	33/58	III-IV	Plasma	Pretreatment, posttreatment	MAPK signaling-targeted drugs	ddPCR	BRAF^V600E^, NRAS^Q61^, TERT^PROM^	NA	21/12^m^ (BRAF^V600E^), 21/12 (NRAS^Q61^)	21/33 (NRAS^Q61^)	PFS, OS	NA	—	6
Váraljai et al., [23] (validation cohort)	German, Belgium	35	III-IV	Plasma	Pretreatment, posttreatment	Pembrolizumab or nivolumab plus ipilimumab	ddPCR	BRAF^V600E^, NRAS^Q61^, TERT^PROM^	NA	20/15^m^	NA	PFS	NA	—	6
Tan et al., [13] (MRV)	Australia	81/133	III	Plasma	Pretreatment, posttreatment	Operation alone or in combination with adjuvant immune checkpoint inhibitor^*∗*^	ddPCR (bio-rad)	BRAF^V600E/V600K^, NRAS^Q61L/Q61K/Q61R^, TP53^R248W^, KIT^L576P^, TERT^C250T/C228T^	≥1 copy of mutant DNA detected in both duplicate reactions	21/37 (pretreatment), 13/39 (posttreatment)	21/58 (pretreatment), 13/52 (posttreatment)	OS	20 months (median)	—	8
Seremet et al., [15]	Belgium	63/85	III-IV	Plasma	Pretreatment, posttreatment^*∗*^	Anti-PD1 therapy pembrolizumab	ddPCR (Bio-Rad) and idylla platform (Biocartis)	BRAF^V600^, NRAS^Q61/G12/G13^	NA	35/28	28/63	PFS, OS	84 weeks (median)	ctDNA, LDH, CRP, number of tumor sites and ECOG variables	7
Forthun et al., [16]	Norway	26	IV	Plasma	Pretreatment, posttreatment	Bevacizumab treatment	ddPCR (Bio-Rad)	NRAS^Q61/G12^, BRAF^V600^, TERT^C288T/C250T^*∗*^^	>2 droplets positive for the mutation assay	NA	23/26 (NRAS^Q61/G12^, BRAF^V600^)	PFS, OS	6 months	ctDNA, LDH	7
Salemi et al., [24]	Italy	26/28	IV (1 case of unknown)	Serum	Pretreatment, posttreatment^*∗*^	BRAF inhibitors alone or in combination with MEK inhibitors	ddPCR (Bio-Rad)	BRAF^V600E^	≥3 FAM/HEX-positive droplets	11/15	11/26	PFS, OS	NA	—	6
McEvoy et al., [25]	Australia	32	IV	Plasma	Pretreatment	Targeted therapy and/or immunotherapy	ddPCR (Bio-Rad)	BRAF, NRAS, KIT	NA	23/9	23/32	PFS	64.4 weeks (median)	ctDNA, age, sex, ECOG, MTB$, stage	7
Lu et al., [26] (chemotherapeutic drug)	12 countries^b^	258/338	IIIC-IV	Plasma	Pretreatment, posttreatment^*∗*^	Chemotherapeutic drug dacarbazine	ddPCR (Bio-Rad)	BRAF^V600^	≥10 copies	172/86	172/258	OS	9.5 months (median)	—	7
Lu et al., [26] (BRAF inhibitor)	12 countries^b^	293/337	IIIC-IV	Plasma	Pretreatment, posttreatment^*∗*^	BRAF inhibitor vemurafenib	ddPCR (Bio-Rad)	BRAF^V600^	≥10 copies	188/105	188/293	OS	12.5 months (median)	—	7
Lee et al., [14]	UK	161	II-III	Plasma	Pretreatment (after operation)	Bevacizumab versus placebo	ddPCR (Bio-Rad)	BRAF^V600E^, NRAS^Q61K/Q61L^	≥1 copy of mutant DNA detected	19/142	19/161	OS	5 years (median)	ctDNA, ECOG, stage	7
Herbreteau et al., [27]	France	53	IIIC-IV	Plasma	Pretreatment, posttreatment^*∗*^	Nivolumab alone or in combination with ipilimumab	dPCR (The QuantStudio 3D digital PCR system)	BRAF^V600E/V600K^, NRAS^Q61K/Q61R/Q61L/G12D^	≥8 mutated copies/mL	30/23	23/53	PFS, OS	6.8 months (median)	—	7
Gonzalez-Cao et al., [28]	Spain	58/66	IV (6 case of unknown)	Serum and plasma	Pretreatment, posttreatment^*∗*^	BRAFi/MEKi, chemotherapy, ipilimumab, palliative	PNA probe-based TaqMan assay	BRAF^V600^	NA	38/20, 15/43^#^	38/58	PFS, OS	NA	—	6
Lee et al., [29]	Australia	76	IV	Plasma	Pretreatment, posttreatment^*∗*^	Pembrolizumab or nivolumab monotherapy, or in combination with ipilimumab	ddPCR (Bio-Rad)	BRAF^V600E/V600K/L597Q/L597R/G464E/G466E^, NRAS^Q61H/Q61K/Q61L/Q61R^, KIT^K642E^	≥3 positive mutant droplets	36/40 (pretreatment), 58/18 (posttreatment)^*∗*^	40/76 (pretreatment), 18/76 (posttreatment)^*∗*^	PFS, OS	17.5 months (median)	ctDNA, LDH, disease volume, ECOG and AJCC tumour stage variables	8
Santiago-Walker et al., [30] (BREAK-3)	12 countries^b^	170/250	III-IV	Plasma	Pretreatment	BRAF inhibitor dabrafenib or DTIC^*∗*^	BEAMing technology	BRAF^V600E/V600K^	Multiple determination methods	137/33	137/170	PFS, OS	4.9 months (median)	—	8
Santiago-Walker et al., [30] (BREAK-MB)	6 countries^a^	54/172	IV	Plasma	Pretreatment	BRAF inhibitor dabrafenib	BEAMing technology	BRAF^V600E/V600K^	Multiple determination methods	40/14	40/54	PFS, OS	4 months	—	8
Santiago-Walker et al., [30] (METRIC)	Multi- countries^b^	169/322	IIIC-IV	Plasma	Pretreatment	MEK inhibitor trametinib or chemotherapy^*∗*^	BEAMing technology	BRAF^V600E/V600K^	Multiple determination methods	124/45	124/169	PFS, OS	NA	—	8
Gray et al., [12] (targeted therapy)	Australia	29	IV	Plasma	Pretreatment, posttreatment^*∗*^	MAPK inhibiting therapies	ddPCR (Bio-Rad)	BRAF^V600E/V600K^, NRAS^Q61R/K^	>1 copy per ml	17/12^#^	NA	PFS	NA	ctDNA levels, age, sex, metastatic disease stage and LDH status	6
Gray et al., [12] (immunotherapy)	Australia	19	IV	Plasma	Pretreatment, posttreatment^*∗*^	Immunotherapies	ddPCR (Bio-Rad)	BRAF^V600E/V600K^, NRAS^Q61R/K^	>1 copy per ml	15/4^#^	NA	PFS	NA	—	6
Board et al., [17]	10 countries^b^	45/126	III-IV	Serum	Pretreatment	MEK inhibitor vs TMZ	ARMS allele-specific PCR	BRAF^V600^	Only if all three replicates were positive	25/20	25/45	PFS	NA	—	6
Shinozaki et al., [31] (biochemotherapy)	USA	20/50	IV	Serum	Posttreatment, pretreatment^*∗*^	Biochemotherapy	Real-time PCR	BRAF^V600E^	The amount of target mutant DNA contained in 1 *µ*g/mL MA genomic DNA	8/12	8/20	OS	NA	—	6

Abbreviations: ddPCR, droplet digital PCR; dPCR, digital PCR; PNA, peptide-nucleic acid; BEAMing technology, beads, emulsion, amplification and magnetic technology; ARMS allele-specific PCR, amplification refractory mutation system allele-specific PCR; PFS, progression-free survival; OS, overall survival; NA, not available; NOS, Newcastle–Ottawa Quality Assessment Scale. ^*∗*^The data were not included in the summary analysis because no prognostic data were available. ^#^The data were grouped into high and low or undetectable ctDNA. ^m^The data were grouped into increase and decrease ctDNA. ^a^Australia, Canada, France, Germany, Italy, and the USA. ^b^The original study did not list specific country names.

## Data Availability

The data supporting this meta-analysis are from previously reported studies, which have been cited. The processed data used to support the findings of this study are included within the article.

## Supplementary Materials

Supplementary Materials
*Table S1.* Heterogeneity analyses: subgroup analysis of PFS and OS (baseline detectable vs. undetectable).
