# Supplementary material for: Prognostic Value of ctDNA Mutation in Melanoma: A Meta-Analysis
Source: J Oncol. 2021 May 4;2021:6660571. doi: 10.1155/2021/6660571 (PMC8116156; doi:10.1155/2021/6660571)
Supplement: Supplementary Materials — Table S1. Heterogeneity analyses: subgroup analysis of PFS and OS (baseline detectable vs. undetectable). [file 6660571.f1.docx]

| **Table S1:Heterogeneity analyses: Subgroup analysis of PFS and OS (baseline detectable vs. undetectable).** | | | | | | | | | |
| --- | --- | --- | --- | --- | --- | --- | --- | --- | --- |
| Variables | OS |  |  |  |  | PFS |  |  |  |
|  | No. of studies/results | HR [95% CI] | P-value | I^2^ (%) |  | No. of studies/results | HR[95% CI] | P-value | I^2^ (%) |
| **Overall** | 10 | 1.97[1.64,2.36] | <0.00001 | 0 |  | 9 | 1.41[0.84,2.37] | 0.19 | 55 |
| **Target gene** |  |  |  |  |  |  |  |  |  |
| BRAF^V600^ | 8 | 1.90[1.58,2.29] | <0.00001 | 0 |  | 7 | 1.02[0.72,1.44] | 0.92 | 4 |
| NRAS^Q61^ | 1 | 4.08[1.57,10.60] | / | / |  | 1 | 3.18[1.31,7.72] | / | / |
| multiple genes | 1 | 2.90[0.52,16.02] | / | / |  | 1 | 7.66[1.76,33.29] | / | / |
| **Sample origin** |  |  |  |  |  |  |  |  |  |
| Plasma | 8 | 1.98[1.65,2.38] | <0.00001 | 0 |  | 6 | 1.62[0.86,3.06] | 0.14 | 65 |
| Serum | 1 | 1.35[0.14,13.37] | / | / |  | 2 | 0.76[0.33,1.72] | 0.51 | 20 |
| Plasma and serum | 1 | 0.26[0.01,9.55] | / | / |  | 1 | 1.12[0.14,8.91] | / | / |
| **Method** |  |  |  |  |  |  |  |  |  |
| ddPCR | 6 | 2.05[1.69,2.49] | <0.00001 | 0 |  | 4 | 2.43[0.87,6.76] | 0.09 | 62 |
| Others | 4 | 1.41[0.82,2.43] | 0.21 | 0 |  | 5 | 1.04[0.71,1.51] | 0.86 | 25 |
| **The data source** |  |  |  |  |  |  |  |  |  |
| Data directly provided by the study | 4 | 2.10[1.73,2.57] | <0.00001 | 0 |  | 2 | 4.02[1.88,8.59] | 0.0003 | 1 |
| Data extracted from the survival curve | 6 | 1.36[0.85,2.15] | 0.2 | 0 |  | 7 | 1.02[0.72,1.44] | 0.92 | 4 |
|  |  |  |  |  |  |  |  |  |  |
| Abbreviations: ddPCR, droplet digital PCR; PFS, progression-free survival; OS, overall survival. | | | | | | |  |  |  |
